# Metformin Exhibits an Attractive Antineoplastic Effect on Human Endometrial Cancer by Regulating the Hippo Signaling Pathway

**DOI:** 10.1155/2022/5824617

**Published:** 2022-08-11

**Authors:** Meng Wang, Qiulin Zhang, Yuehan Li, Lei Jin, Zishui Fang

**Affiliations:** ^1^Reproductive Medicine Center, Tongji Hospital, Tongji Medical College, Huazhong University of Science and Technology, Wuhan, China; ^2^Department of Reproductive Medicine, Maternal and Child Hospital of Hubei Province, Tongji Medical College, Huazhong University of Science and Technology, Wuhan, China

## Abstract

Metformin, the first-line oral antidiabetic medicine, has shown great antineoplastic potential in various cancer types, despite an unclear mechanism. This study aimed to elucidate the possible mechanism of metformin as a chemotherapy agent with less reproductive and genetic toxicity in human endometrial cancer. The type I endometrial carcinoma cell lines Ishikawa and RL95-2 were treated with metformin. Cell functions, such as proliferation, migration, and invasion, were analyzed. Flow cytometry was performed for cell cycle and apoptosis analyses. Simultaneously, RT-qPCR and western blotting were performed to explore the possible mechanism. Moreover, YAP1 knockout Ishikawa cells were established via lentivirus to demonstrate the underlying mechanism. The results showed that metformin mediated Ishikawa and RL95-2 cell growth inhibition in a dose- and time-dependent manner. The IC50 values of metformin in Ishikawa and RL95-2 cells were 10 mM and 8 mM, respectively. The migration and invasion abilities were also inhibited in the metformin-treated group using wound healing assays and transwell migration and invasion assays, and Ishikawa and RL95-2 cells were arrested in the G1 or G2 phase, respectively. Moreover, the cell proportions of cells in both early and late apoptosis stages were dramatically elevated when treated with metformin, as was the ratio of Bax/Bcl-2 expression. Additionally, the expression levels of YAP1 mRNA and protein in the treatment group were much lower than those in the control group. The cellular behaviors of YAP1 knockout Ishikawa cells were similar to those in the metformin-treated group. Our results demonstrated that it is an attractive alternative to cytotoxic chemotherapy in human endometrial cancer, and YAP of the Hippo pathway may be a potential molecular target. This study provides novel ideas for the adjuvant therapy of endometrial cancer patients, especially for women with strong fertility desires and demands.

## 1. Introduction

Endometrial cancer is the most frequently occurring gynecologic malignancy in Western countries. It has been reported that since 2008, the incidence of endometrial cancer has increased by 21% in the United States, as well as doubling the death rate over the past two decades [1]. Although the utilization of nanotechnology has greatly impacted cancer therapeutics [2–5], chemotherapy is still a common adjuvant therapy for advanced or recurrent endometrial cancer; however, the devastating blow to the female reproductive system makes traditional platinum-based chemotherapy unsuitable for women who wish to preserve fertility [6]. Therefore, the development of new chemotherapeutic agents that have comparable efficacy and fewer adverse effects on the reproductive system for endometrial cancer patients of reproductive age is needed.

The Hippo signaling pathway is an evolutionarily conserved signaling transduction and regulator involved in cell differentiation, tissue regeneration, organ development, and immune modulation [7]. The core of this signaling cascade comprises kinases such as large tumor suppressor 1/2 (LATS1/2) and the transcriptional coactivator Yes-associated protein (YAP) [8]. Phosphorylation of YAP activated by LATS kinases results in their cytoplasmic retention and degradation, which causes the failure of their binding with transcriptional enhanced associate domains (TEADs) and other potential transcription factors in the nucleus to repress gene transcription [9, 10]. Recently, an increasing number of studies on human carcinomas have validated the significant role of the Hippo pathway in tumorigenesis and tumor progression [11]. YAP is also upregulated and closely associated with the degree of malignancy and prognosis in several gynecologic cancers, including cervical [12], ovarian [13], and endometrial cancer [14]. Thus, cumulative evidence has demonstrated YAP as a possible new molecular target for cancer therapy [15–17], and it is practical to seek an alternative Hippo-targeted agent with limited reproductive and genetic toxicity for endometrial cancer.

Metformin, the most widely used antidiabetic drug [18], has been proposed to have antineoplastic properties against certain types of cancers, including gynecological cancers [19, 20]. Metformin has been reported to attenuate the stemness of glioma cells [21] and suppress cell proliferation and invasion in non-small cell lung cancer [22] by downregulating the expression of YAP. Considering the high expression of YAP in endometrial cancer, it is reasonable to speculate the possible link between the inhibitory effect of metformin on endometrial cancer and the Hippo signaling pathway. Moreover, metformin has also exhibited reproductive endocrine modulatory properties [23, 24], which makes metformin an attractive alternative to cytotoxic chemotherapy.

The present study aims to explore the antineoplastic effect of metformin on endometrial cancer *in vitro* and the role of the Hippo signaling pathway in the process.

## 2. Methods

### 2.1. Cell Culture and Transfection

Human type I endometrial carcinoma Ishikawa and RL95-2 cells were previously donated by the Cancer Biology Research Center of Tongji Hospital, Tongji Medical College, Huazhong University of Science and Technology. Cells were cultured in Dulbecco's modified Eagle medium (DMEM)-high glucose (HyClone, United States) containing 10% (*v/v*) fetal bovine serum (FBS, Gibco, United States), 100 U/mL penicillin (Boster, China), and 100 *μ*g/mL streptomycin (Boster, China) at 37°C in a humidified atmosphere with 5% CO_2_ in an incubator (MCO-18AC, Panasonic, Japan). Metformin (S1950, Selleck, China) was dissolved in ddH_2_O and then diluted to a suitable concentration. *YAP1*-knockout (YAP1-KO) lentivirus (LV-YAP1-KO) based on the CRISPR/Cas9 (Clustered Regularly Interspaced Short Palindromic Repeats/CRISPR-associated 9) system was purchased from Genechem, China. Ishikawa cells were transfected with LV-YAP-KO using HitransG P (Genechem, China). Following selection with 5 *μ*g/ml puromycin (CL13900, Selleck, China) for 72 h, single-cell clones were sorted, expanded, and validated as YAP1-KO Ishikawa cells by western blotting and reverse transcription-quantitative polymerase chain reaction (RT-qPCR) analysis.

### 2.2. Cell Viability Assay

Cell function assays included cell viability assays, flow cytometry analysis, wound healing assays, transwell migration assays, and *in vitro* transwell invasion assays. Cell viability was evaluated by CCK-8 assay using a CCK-8 kit purchased from MCE, China, following the manufacturer's instructions. Briefly, cells were incubated with various concentrations of metformin for different times. Then, 10 *μ*L CCK-8 reagent was added to the medium 2 h before OD450 measurement using a spectrophotometer (BioTek, United States).

### 2.3. Flow Cytometry Assay

The cell cycle was analyzed using flow cytometry. Cell pellets were resuspended in precooled 90% ethanol and then incubated at −20°C overnight. After washing and incubating in a water bath, the cells were stained with propidium iodide (PI, BD Biosciences, United States) for 20 min in the dark before flow cytometry analysis. Dual staining was performed for the apoptosis assay using an Annexin V-FITC/PI kit (Cat. No. 556547, BD Biosciences, United States). Cells stained with FITC-conjugated Annexin V and PI were measured by flow cytometry.

### 2.4. Wound Healing Assay

Well-spread cells in 24-well plates were scratched by a pipette tip after FBS starvation for 12 h and then observed every 24 h using an inverted microscope (Axio Observer A1, Carl Zeiss, Germany). The relative wound areas were quantified and calculated by ImageJ (NIH, United States).

### 2.5. Transwell Migration and Invasion Assay

For the transwell migration and invasion assays, 10,000/mL cells in 100 *μ*L FBS-free medium were seeded in the top chambers of transwells (for the migration assay, Corning, United States) and Matrigel-coated transwells (for the invasion assay, Corning, United States), and the medium on the bottom was supplemented with 20% FBS. After 48 h of culture, cells in the chambers were fixed with 4% paraformaldehyde, stained with crystal violet (Servicebio, China), and then counted under a 20× microscope (Axio Observer A1, Carl Zeiss, Germany).

### 2.6. Western Blotting Analysis

The concentrations of total protein extracted from the cells were quantified using a BCA assay (MCE, China). After separation using 10% SDS-PAGE, the proteins were transferred to PVDF membranes (Millipore, United States) and then incubated with the corresponding primary antibody and horseradish peroxidase-conjugated secondary antibody according to the manufacturer's instructions. Protein visualization was performed using an ECL kit (HYK1005, MCE, China). Protein expression was quantified using ImageJ software (NIH, United States). Anti-LAST1 (17049-1-AP), anti-Bcl-2 (12789-1-AP), anti-Bax (1 : 2000, 50599-2-Ig), anti-GAPDH (10494-1-AP), and anti-*β*-actin (66099-1-Ig) were purchased from Proteintech, China. Anti-YAP1 (#14074) and anti-phospho-YAP1 (#13008) were purchased from CST, United States. The secondary antibodies (1 : 2000) were purchased from Servicebio, China. The antibodies mentioned above were diluted at a ratio of 1 : 1000 unless stated otherwise.

### 2.7. RT-qPCR Analysis

Total RNA was isolated from the cells using NucleoZol reagent (Macherey-Nagel, Germany). Then, 500 ng of total RNA was used to synthesize cDNA using a reverse transcription kit (RR036 A, Takara, Japan), and qPCR was performed using TB Green *Premix Ex Taq* (RR420 A, Takara, Japan) following the manufacturer's instructions. The mRNA expression was calculated using the 2^−ΔΔCt^ method and normalized to GAPDH. The sequences of the primers were as follows: YAP1, 5′-ACTGGCTACGCAGGGCTA-3′ (F), 5′-TTTGAGTCCCACCATCCTGCTCCAG-3′ (R), GAPDH: 5′-TGAGTCCTTCCACGATACCAA-3′ (F), 5′-AGTCTTCTGGGTGGCAGTGA-3′ (R).

### 2.8. Statistical Analysis

Statistical analyses were performed by using SPSS version 26.0 (IBM, United States), and data visualization was carried out using GraphPad Prism 8.0 (GraphPad Software Inc., United States). All data are presented as the mean ± standard deviation (SD). Student's independent *t*-test was used for comparisons between two groups, and analysis of variance (ANOVA) was used for comparisons among three groups. Two-tailed hypothesis tests were performed. *P* < 0.05 was considered significant. Each experiment was performed in triplicate and repeated at least three times independently.

## 3. Results

### 3.1. Metformin Inhibited Cell Proliferation and Induced Cell Cycle Arrest in Endometrial Carcinoma Cells

**Figure 1:**
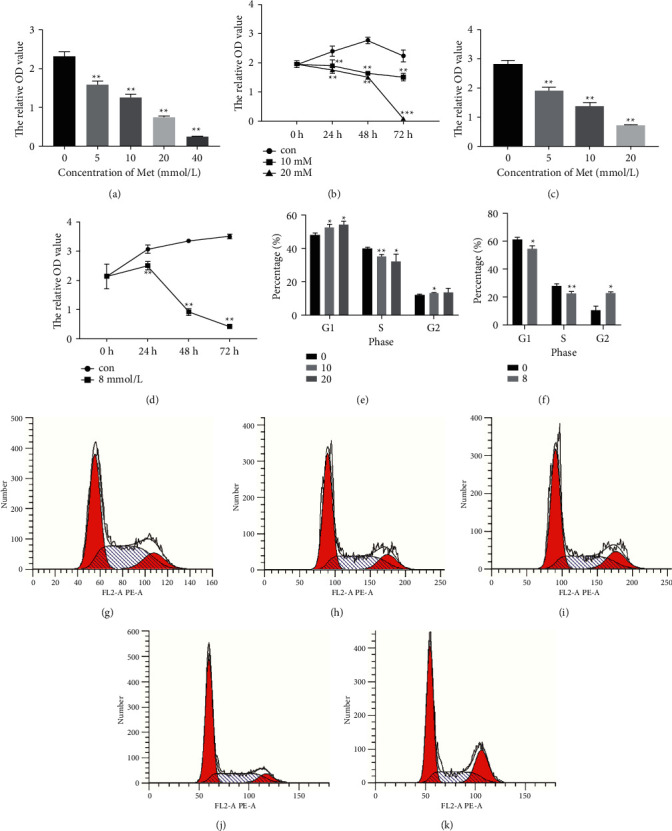
Metformin inhibited cell proliferation and induced cell cycle arrest in endometrial carcinoma cells. Ishikawa and RL95-2 cells were incubated with metformin at different concentrations (0, 5, 10, 20, and 40 mM for Ishikawa cells and 0, 5, 10, and 20 mM for RL95-2 cells), and the cell proliferation ability was detected using CCK-8 assays. (a and b) Cell proliferation inhibition of Ishikawa cells was dependent on the dose and time of metformin exposure. (c and d) Metformin mediated RL95-2 cell growth inhibition in a dose- and time-dependent manner. (e) Compared with the control group (g), 10 mM (h) and 20 mM (i) metformin treatment resulted in cell cycle arrest at the G1 phase in Ishikawa cells. (f) 8 mM (k) metformin was capable of arresting the cell cycle at the G2 phase in PL95-2 cells in comparison with the nontreated group (j). Each experiment was performed in triplicate and repeated at least three times independently. Data are presented as the mean ± SD. ^*∗*^*P* < 0.05; ^*∗∗*^*P* < 0.01; ^*∗∗∗*^*P* < 0.001 versus controls. Met, metformin; Con, control.

Ishikawa and RL95-2 cells were incubated with metformin at different concentrations (0, 5, 10, 20, and 40 mM for Ishikawa cells and 0, 5, 10, and 20 mM for RL95-2 cells), and the cell proliferation ability was detected using CCK-8 assays. As shown in Figure 1(a), with the prolongation of the concentration of metformin treatment, the relative OD value of cells decreased sharply, which indicated that cell proliferation inhibition of Ishikawa was dependent on the dose of metformin exposure. The IC50 of metformin in Ishikawa cells was calculated, and the estimated value was 10 mM. Then, Ishikawa cells were treated with 0, 10, and 20 mM metformin for various times (0, 24, 48, and 72 h), and significant time-dependent inhibition was demonstrated (Figure 1(b)). A similar phenomenon can also be observed in RL95-2 (Figures 1(c) and 1(d)), and the IC50 value of metformin in RL95-2 cells was 8 mM. These results indicated that metformin also mediated RL95-2 cell growth inhibition in a dose- and time-dependent manner. Based on the results of the CCK-8 assays, 48 h was chosen as the treatment time of the agent. To investigate the effect of metformin on endometrial carcinoma cells, the cell cycle was analyzed by flow cytometry. As shown in Figure 1(e), compared with the control group (Figure 1(g)), the proportion of Ishikawa cells in the G1 phase increased significantly, and the ratio of cells in the S phase decreased in the 10 mM (Figure 1(h)) and 20 mM (Figure 1(i)) metformin-treated group. This result demonstrated that metformin exposure resulted in cell cycle arrest at the G1 phase in Ishikawa cells. Similarly (Figure 1(f)), metformin was found to be capable of arresting the cell cycle at the G2 phase in PL95-2 cells (Figure 1(k)) in comparison with the nontreated group (Figure 1(j)).

### 3.2. Metformin Induced Cell Apoptosis in Endometrial Carcinoma Cells

**Figure 2:**
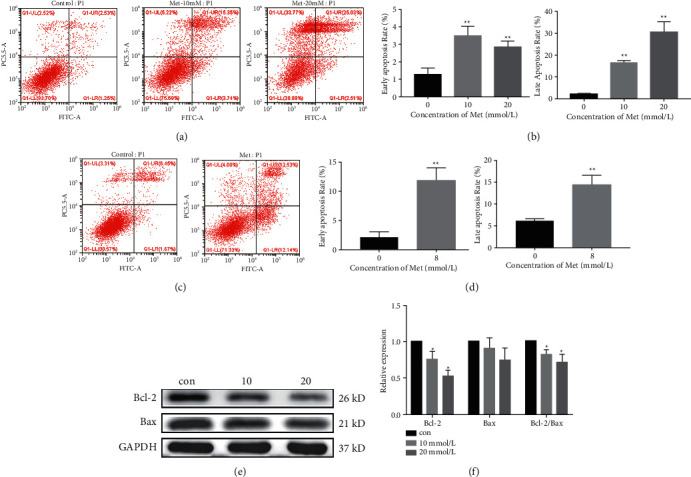
Metformin induced cell apoptosis in endometrial carcinoma cells. Cell apoptosis rates were examined in Ishikawa and RL95-2 cells after 48 h metformin treatment (10 and 20 mM for Ishikawa and 8 mM for RL95-2) by flow cytometry. (a and b) Metformin increased the early and late apoptosis rates in Ishikawa cells, and the majority of apoptotic cells were at the late apoptosis stage. (c and d) The apoptosis percentage of RL95-2 cells increased in the metformin exposure group. (e and f) The ratio of Bcl-2/Bax decreased as the concentration of metformin increased in Ishikawa cells. Each experiment was performed in triplicate and repeated at least three times independently. Data are presented as the mean ± SD. ^*∗*^*P* < 0.05; ^*∗∗*^*P* < 0.01 versus controls. Met, metformin; Con, control.

Cell apoptosis rates were examined in Ishikawa and RL95-2 cells after 48 h metformin treatment (10 and 20 mM for Ishikawa cells, 8 mM for RL95-2 cells) by Annexin V-FITC/PI flow cytometry. FITC + PI- cells were regarded to be in the early stage of apoptosis, and FITC + PI + cells were in the late stage of apoptosis. As shown in Figures 2(a) and 2(b), the proportion of cells in the early and late apoptosis stages was dramatically elevated, and the majority of apoptotic cells were at the late apoptosis stage. The apoptosis percentage of RL95-2 cells increased in the metformin exposure group, despite a comparable ratio of early stage and late stage apoptosis (Figures 2(c) and 2(d)). To further confirm the flow cytometry results, the protein expression levels of Bcl-2 and Bax were tested using western blotting. The ratio of Bcl-2/Bax decreased in Ishikawa cells treated with metformin (Figures 2(e) and 2(f)), which indicated that the mitochondrial apoptosis pathway may be involved in apoptosis of Ishikawa cells induced by metformin.

### 3.3. Metformin Inhibited Cell Migration and Invasion in Endometrial Carcinoma Cells

**Figure 3:**
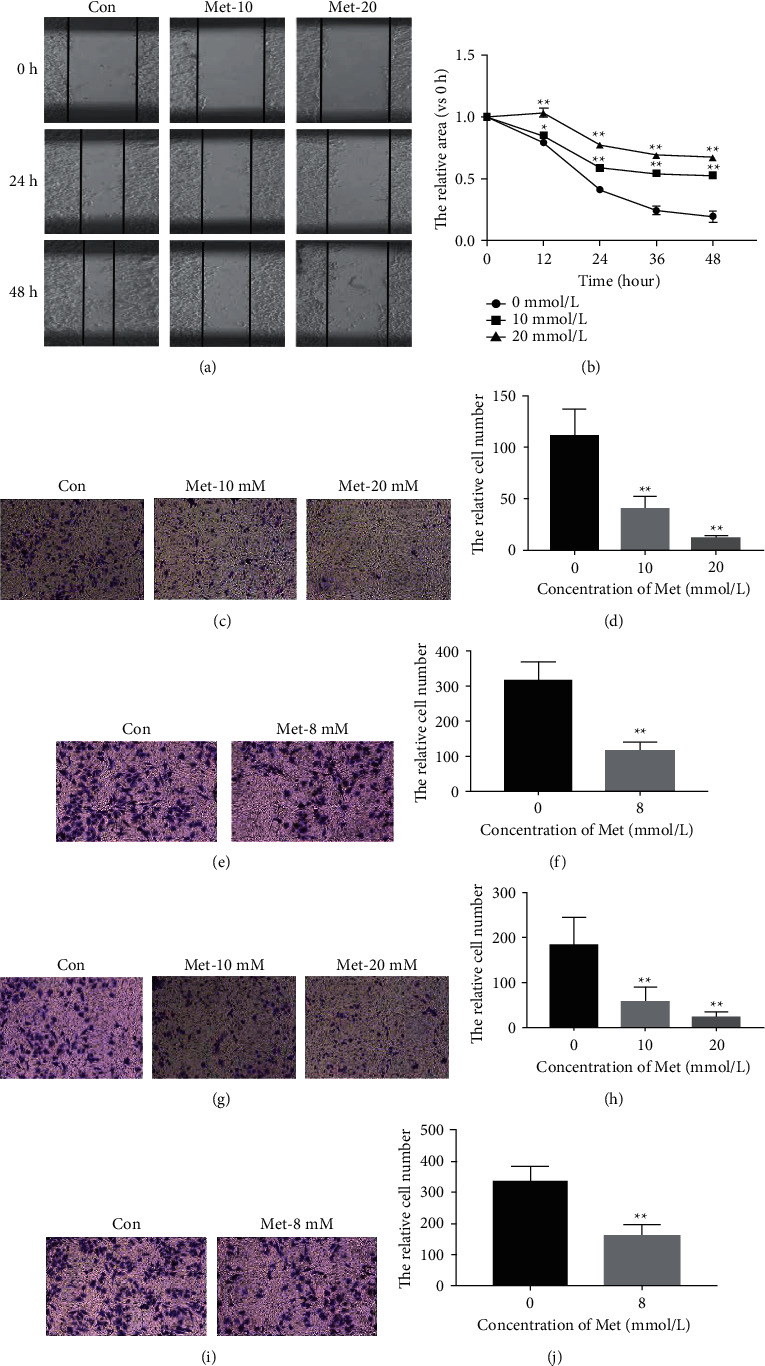
Metformin inhibited cell migration and invasion in endometrial carcinoma cells. Cell migration ability was detected using a wound healing assay and transwell migration assay. The invasion ability was determined using *in vitro* transwell invasion assays. (a and b) The decrease in relative areas between the scratches in the higher concentration metformin treatment group was less evident, which indicated an inhibitory effect of metformin on Ishikawa cell migration. (c and d) Metformin greatly inhibited cell migration in Ishikawa cells. (e and f) The inhibition of cell migration in metformin-treated RL95-2 cells was more significant. (g and h) Cell invasion after exposure to different concentrations of metformin in Ishikawa cells. (i and j) Cell invasion was inhibited by metformin in RL95-2 cells. Each experiment was performed in triplicate and repeated at least three times independently. Data are presented as the mean ± SD. ^*∗*^*P* < 0.05; ^*∗∗*^*P* < 0.01 versus controls. Met, metformin; Con, control.

Ishikawa and RL95-2 cells were treated with metformin (10 and 20 mM for Ishikawa cells, 8 mM for RL95-2 cells) to investigate cell migration and invasion. Since confluence growth was absent in RL95-2 cells, the wound healing assay was only performed in Ishikawa cells. The relative areas between the scratches in the control group were positively correlated with exposure time, and the wound was almost healed after 48 h of treatment. In the metformin treatment group, the speed of wound healing was significantly slower, especially with a higher concentration of metformin, which indicated an inhibitory effect of metformin on Ishikawa cell migration (Figures 3(a) and 3(b)). The results of the transwell migration assay have also corroborated the inhibitory effect of metformin on Ishikawa and RL95-2 cell migration. The number of adherent Ishikawa cells in the control group was 111.1 ± 26.1 at 100× magnification, which was higher than that in the 10 mM metformin group (39.3 ± 13.0) and the 20 mM metformin group (10.9 ± 3.0) (*P* < 0.01), demonstrating that metformin inhibited Ishikawa *in vitro* migration in a dose-dependent pattern (Figures 3(c) and 3(d)). A more obvious inhibition of cell migration was observed in metformin-treated RL95-2 cells (Figures 3(e) and 3(f)). Then, the invasion ability was determined using *in vitro* transwell invasion assays, and the number of penetrating cells reflected the invasive ability of tumor cells. Compared with the control group (183.4 ± 61.9 at 100× magnification), the number of penetrating cells was much lower than that in the 10 mM metformin group (55.7 ± 33.3) and the 20 mM metformin group (20.6 ± 14.0) (*P* < 0.01) (Figures 3(g) and 3(h)). Similar results were also observed in the RL95-2 cells (Figures 3(g) and 3(j)).

The Hippo signaling pathway may be an antitumor target of metformin in endometrial carcinoma cells.

**Figure 4:**
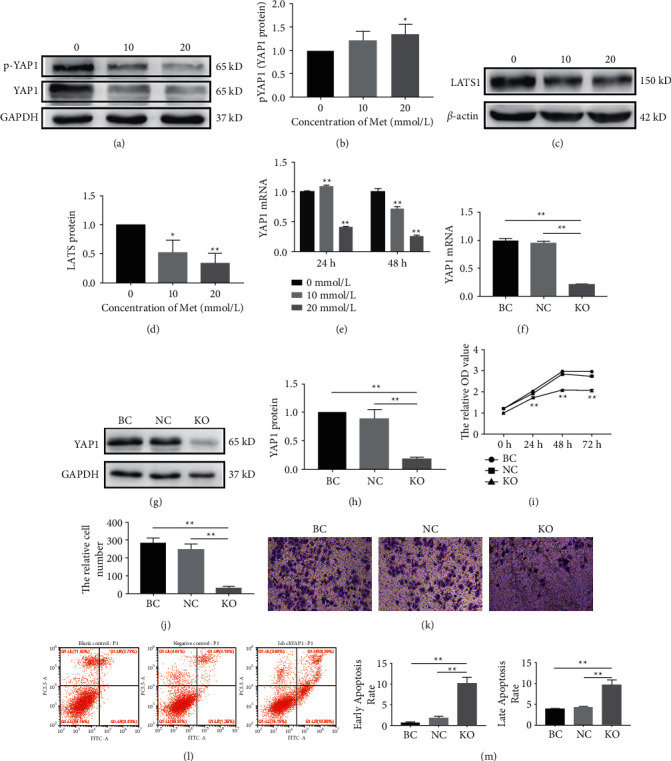
The Hippo signaling pathway may be an antitumor target of metformin in endometrial carcinoma cells. The possible mechanism of the antitumor effect of metformin was explored in Ishikawa cells. (a and b) Protein expression was detected using western blotting, and the relative protein level of p-YAP1/YAP1 increased obviously in the metformin-treated group. (c and d) A dramatic decrease in the protein expression of LATS was observed in the treatment group. (e) The YAP1 mRNA level showed a consistent trend with the protein level. YAP1 knockout (YAP1-KO) Ishikawa cells were obtained using the CRISPR/Cas9 technique. (f–h) Both the mRNA and protein levels of YAP1 were examined in YAP1-KO Ishikawa cells. (i) The cell proliferation ability of YAP1-KO Ishikawa decreased significantly compared with that of the control groups. (j and k) The transwell invasion assay also indicated a decreased invasion ability in YAP1-KO Ishikawa cells. (l and m) Knockout of YAP1 in Ishikawa cells sharply increased the percentage of apoptotic cells. Each experiment was performed in triplicate and repeated at least three times independently. Data are presented as the mean ± SD. ^*∗*^*P* < 0.05; ^*∗∗*^*P* < 0.01 versus controls. Met, metformin; BC, blank control; NC, negative control; KO, knockout.

Ishikawa cells were treated with various concentrations of metformin (0, 10, and 20 mM) for 48 h to explore the possible mechanism of the antitumor effect of metformin. The mRNA and protein expression of YAP1 and LATS in Ishikawa cells were detected using qRT-PCR and western blotting. The relative protein level of p-YAP1/YAP1 increased obviously in the metformin-treated group (Figures 4(a)–4(b)). Additionally, a dramatic decrease in the protein expression of LATS was observed in the treatment group (Figures 4(c) and 4(d)). Moreover, the YAP1 mRNA level showed a consistent trend with the protein level (Figure 4(e)). These results indicated that metformin can activate the Hippo signaling pathway in Ishikawa cells. The efficiency of YAP1 knockout in Ishikawa cells was confirmed using qRT-PCR and western blotting, and our results showed a dramatic decrease in the mRNA and protein expression of YAP1 in the YAP1-KO group compared with the BC and NC groups (Figures 4(f)–4(h)). The cell proliferation ability of YAP1-KO Ishikawa cells was investigated using CCK-8 assays, and the lack of YAP1 inhibited the cell proliferation of Ishikawa cells (Figure 4(i)). Additionally, a transwell invasion assay was performed to detect the effect of YAP1 deletion on *in vitro* invasion in Ishikawa cells, and the results demonstrated the obvious inhibition of invasion in YAP1-KO Ishikawa cells (Figures 4(j) and 4(k)). Equally, the knockout of YAP1 in Ishikawa cells also sharply increased the percentage of apoptosis in both the early and late apoptosis stages (Figures 4(l) and 4(m)).

## 4. Discussion

**Figure 5:**
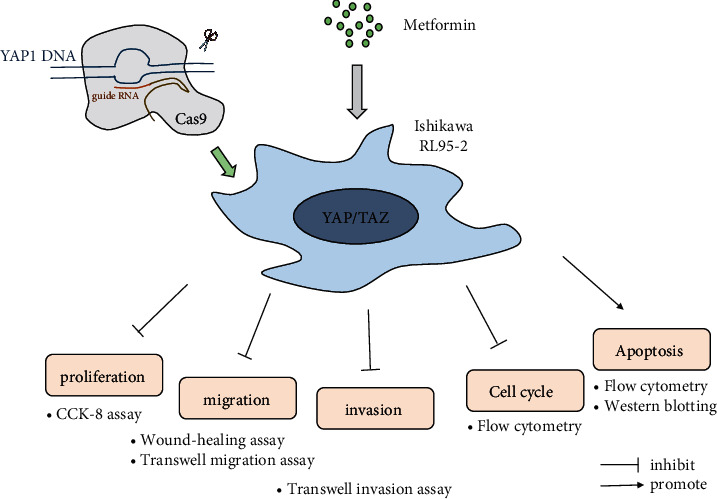
The flow chart of this study.

In the current study, we found that metformin was able to inhibit cell proliferation, migration, and invasion, as well as induce cell cycle arrest and apoptosis in human endometrial cancer cells. Moreover, YAP, the core of the Hippo signaling pathway, might be a potential target for the antineoplastic therapy with metformin in endometrial cancer (Figure 5).

First, Ishikawa and RL95-2 cells were treated with various concentrations of metformin at different times and analyzed by CCK-8 assay to seek a suitable treatment concentration and time. Cell cycle arrest in G1 and G2 phases can be observed by flow cytometry, and metformin is likely to interfere with the process of DNA replication. The results of Annexin V/PI dual staining flow cytometry also proved that metformin induced both early and late stages of apoptosis, which was further reinforced by western blotting. Moreover, the decrease in Bcl-2/Bax in the metformin-treated group suggested the possible participation of the mitochondrial pathway. In addition, more cell functions were investigated through wound healing tests and transwell analysis, and significant inhibition of cell migration and invasion was observed. The above results indicated the significant suppression effects of metformin on endometrial cancer cells *in vitro*.

Metformin has shown great potential for antidiabetic, antiphlogistic, antilipemic, and antineoplastic properties. It has been reported that metformin reduces the incidence and death rate and improves the prognosis of multiple malignancies, including hepatocellular [25], pancreatic [26], ovarian [19], and cervical carcinoma [20]. However, the possible mechanism remains unclear. Some studies reported that metformin activated the AMP-activated protein kinase (AMPK) signaling pathway [27], inhibited the expression of the proto-oncogene human epidermal growth factor receptor 2 (HER-2) [28], and resulted in cell cycle arrest and proliferation inhibition [29]. In other studies, metformin has also been proposed to downregulate YAP expression to attenuate cell proliferation and invasion in non-small cell lung cancer [22]. Dysfunction of the Hippo signaling pathway is widely proven to promote tumorigenesis [30], and YAP, as the heart of the Hippo signaling pathway, acts as a coactivator to activate cell proliferation genes, such as connective tissue growth factor (CTGF) and brain-derived neurotrophic factor (BDNF) [9]. In our study, YAP was chosen as the target and knocked out in Ishikawa cells using the CRISPR/Cas9 system, which is an RNA-guided genome editing tool consisting of a Cas9 nuclease and a single-guide RNA (sgRNA). Similar tumor suppression induced by metformin can be observed, which supports the hypotheses that the Hippo signaling pathway is likely to participate in the regulation of cell death and the fate of endometrial cancer cells. It has been reported that YAP/TAZ, the transcriptional regulators of the Hippo pathway, are highly expressed in various cancers, and the elevation of YAP/TAZ levels in endometrial cancer cell lines has been proven to increase cell proliferation, migration, and invasion [31–33]. Moreover, TAZ/YAP was associated with the PI3K/AKT pathway in endometrial cancer, in which the activation of the Hippo pathways reduced the activation of the PI3K/AKT pathway, affecting multiple physiological processes [31, 34]. In addition, YAP can interact with the promoter of IL-6, increasing the transcription level of IL-6, which is significant for the tumorigenesis of endometrial cancer cell lines [32]. In conclusion, the Hippo pathway is a new molecular mechanism for the development of endometrial cancer, providing novel insight into therapeutic strategies targeting the Hippo pathway, in addition to traditional therapeutics.

The Ishikawa and RL95-2 cell lines both originated from type I endometrial cancers, which are hormone-dependent and caused by cumulated estrogen with the lack of the antagonism of progesterone. Type I endometrial cancer is usually well-differentiated adenocarcinoma, normally accompanied by obesity, diabetes, and infertility, and secondary to atypical hyperplasia of the endometrium [35]. Interestingly, type 2 diabetes mellitus (T2DM) is also known to be a key risk factor for endometrial cancer [36], and a meta-analysis involving 21 cohort studies showed an obvious positive relationship between a preexisting history of diabetes mellitus and the incidence of endometrial cancer, despite no significant risk of mortality [37]. Our study confirmed that the first-line antidiabetic metformin for T2DM had potential antineoplastic properties in endometrial cancer, which was also proven in another study [38]. In addition, several studies have emphasized the attentional link between T2DM and endometrial cancer risk and mortality, mainly through obesity and chronic inflammation [39]. Our findings shed light on the other possible connection between these two highly frequent diseases.

Drug safety is always the focal concern of health care providers. Metformin has been proposed to improve the clinical outcomes of fertility in females suffering from polycystic ovary syndrome (PCOS) with insulin resistance [40]. Compared with traditional chemotherapy agents such as cisplatin, metformin showed less reproductive and genetic toxicity [23]. However, it should be noted that the concentrations of metformin used in our *in vitro* studies, with an IC50 of 8 to 10 mM, were much higher than those in animals, which were between 40 and 70 *μ*M of the plasma concentration in veins after the administration of a therapeutic dose [41]. A similar study also revealed that metformin at an antidiabetic dose still exhibited an antineoplastic effect on cancer cells [38], but it was only a preclinical study and the conclusion still needs to be discussed and confirmed. Therefore, a safe and effective dose of metformin must be explored in a well-established endometrial cancer animal model in the future.

There were still limitations in this study. We only detected the expression of a few proteins and mRNAs involved in apoptosis and the Hippo pathway, and the exploration of the mechanism was not comprehensive and deep enough. In addition, our conclusions were based on *in vitro* cell experiments, and more animal studies are needed to reinforce our current results and conclusions. Moreover, the functional routes of metformin in endometrial cancer are complicated and cross-linked, and the Hippo signaling pathway may not be unique. The exact signaling pathway network remains to be explored. The lack of comparison of metformin and traditional antitumor drugs such as paclitaxel can be another limitation. Whether metformin can achieve a similar antitumor effect as other drugs is still unclear. More mechanistic studies with a better design are needed to investigate the anticancer effect of metformin on endometrial cancer in the future.

## 5. Conclusions

In conclusion, metformin treatment in human type I endometrial carcinoma Ishikawa cells and RL95-2 cells can inhibit proliferation, migration, and invasion, block the cell cycle, and promote apoptosis, demonstrating that metformin is an attractive alternative to cytotoxic chemotherapy in human endometrial cancer and that YAP of the Hippo pathway may be a potential molecular target. This study provides novel ideas for the adjuvant therapy of endometrial cancer patients, especially for women with strong fertility desires and demands.

## Data Availability

All data generated or analyzed during this study are included in this published article.
